# Transcriptional expression of 8 genes predicts pathological response to first-line docetaxel + trastuzumab-based neoadjuvant chemotherapy

**DOI:** 10.1186/s12885-015-1198-9

**Published:** 2015-03-24

**Authors:** Esther Schmitt, Frédérique Végran, Sandy Chevrier, Laura Burillier, Muriel Cadouot, Sarab Lizard-Nacol, Bruno Coudert, Pierre Fumoleau, Laurent Arnould, Romain Boidot

**Affiliations:** 1 Molecular Biology Unit, Centre Georges-François Leclerc, 1, rue du Professeur Marion, Dijon, 21079 Cedex France; 2 Department of Oncology, Centre Georges-François Leclerc, 1, rue du Professeur Marion, Dijon, 21079 Cedex France; 3 Pathology Unit, Centre Georges-François Leclerc, 1, rue du Professeur Marion, Dijon, 21079 Cedex France; 4 Platform for Transfer to Cancer Biology, Centre Georges-François Leclerc, 1, rue du Professeur Marion, Dijon, 21079 Cedex France; 5 U866 Inserm, 7, boulevard Jeanne d’Arc, Dijon, 21000 France

**Keywords:** HER2, Breast, Response prediction, Trastuzumab + docetaxel, First-line neoadjuvant treatment

## Abstract

**Background:**

Overexpression of HER2 is observed in 20 to 30% of breast carcinomas. The use of trastuzumab has improved the treatment of these patients, especially when it is associated with docetaxel. To optimize the use of this treatment, it seems important to select putative complete responders before treatment administration.

**Methods:**

In this study, we analyzed by quantitative PCR the expression of 28 genes in HER2-overexpressing tumors treated with trastuzumab + docetaxel-based chemotherapy. We then correlated their expression profile with those of trastuzumab-sensitive and resistant cell lines to classify tumors as having a sensitive (pCR) or resistant (non-pCR) profile. Finally, we used public datasets from the GEO website to validate the reduced gene-expression profile obtained.

**Results:**

We identified an 8-gene-expression combination that predicted the response to treatment with an accuracy of 76%. Based on public microarray data, we showed that the expression profile was specific to first-line trastuzumab + docetaxel-based treatment with an accuracy of 85%.

**Conclusions:**

Our results showed that by profiling the expression of 8 genes it was possible to predict the response to first-line trastuzumab + docetaxel-based chemotherapy. The use of cancer cell lines as the reference allowed a proper fit with the specificity of different tissues, such as lung or gastric cancers, which could also be eligible to concomitant HER2 inhibition by treatment with trastuzumab or tyrosine kinase inhibitors and docetaxel.

**Electronic supplementary material:**

The online version of this article (doi:10.1186/s12885-015-1198-9) contains supplementary material, which is available to authorized users.

## Background

Breast cancer is the leading cause of death by cancer in women in industrialized countries. The amplification and overexpression of human epidermal growth factor receptor 2 (HER2) is observed in 20–30% of invasive breast cancers. For locally-advanced, HER2-overexpressing breast cancer, docetaxel + trastuzumab-based neoadjuvant chemotherapy has been shown to achieve promising efficacy, with a good pathological complete response (pCR) rate, while being well tolerated in women with stage II or III HER2-positive breast cancer [1,2]. Women who achieve a pCR have significantly improved survival [3], but only 50% of patients with HER2-positive tumors treated with trastuzumab have pCR.

In a previous study [4], we showed that a 28-gene signature dichotomized responses to trastuzumab + docetaxel-based regimens. In the present study, we used real-time quantitative PCR to analyze the expression of these 28 genes in 45 frozen HER2+++ tumors and 6 mammary cancer cell lines that were sensitive or resistant to trastuzumab (Additional file 1). Next, we used public datasets (GSE37946 [5], GSE22358 [6], and GSE42822 [7]) to test the prediction capacity of the refined signature in different treatment regimens.

## Methods

### Patients and samples

We retrospectively analyzed 45 frozen HER2+++ tumors (Table 1). In addition, we also studied 34 FFPE HER2+++ tumors (Table 1). The study was conducted in accordance with the Declaration of Helsinki and approved by an Ethics Committee, the Comité Consultatif de Protection des Personnes en Recherche Biomédicale de Bourgogne. Written informed consent was obtained from all patients before enrollment. RNA was extracted from frozen samples as described previously [4] and from FFPE samples with the RNeasy FFPE kit (Qiagen) by following manufacturer’s protocol.

**Table 1:** **Demographic data of patients analyzed**

Clinical parameters	Frozen samples n = 45	FFPE samples n = 34
**Age**		
≤50	28	24
>50	17	10
**Hormone receptors**		
Estrogen Receptors -	18	15
Estrogen Receptors +	27	19
Progesterone Receptors -	24	17
Progesterone Receptors +	21	17
**Grade**		
1	4	2
2	23	19
3	16	11
Not available	2	2
**Tumour size**		
2-4 cm	23	18
>4 cm	7	5
Not available	15	11
**Pathological response**		
pCR	18	12
non-pCR	27	22

Trastuzumab-sensitive cell lines BT474, HCC2218, UACC-812, and resistant cell lines HCC1419, HCC1954, and HCC1569 were obtained from ATCC and cultured in accordance with the supplier’s instructions. The treatment of cells before RNA extraction was identical to that for patients’ tumors.

### Gene expression analysis

The transcriptional expression of the 28 genes was studied by real-time quantitative PCR thanks to Taqman Gene Expression Assays: PEX19 (Hs00267867), PSMD11 (Hs00160660), SENP8 (Hs00744981), PRKACA (Hs00427274), CTNS (Hs00191849), NFE2L1 (Hs00231457), PPP2CA (Hs00427259), SENP7 (Hs00221046), SYNCRIP (Hs03044160), CEP89 (Hs01071366), SLC30A6 (Hs00215827), LAMA3 (Hs00165042), STX1A (Hs00270282), GPR22 (Hs01127309), GRHL2 (Hs00227745), DERL1 (Hs00225583), FAM114A2 (Hs03837084), PITPNA (Hs00737576), CDC14A (Hs00185432), SLC35A4 (Hs00365408), KIAA1549 (Hs00860114), LOC158402 (Hs00327489), ZNF146 (Hs00173196), C5orf3 (Hs00218834), WEE1 (Hs01119384), P2RX1 (Hs00175686), MFSD6 (Hs00214462), except for HNC20 transcript (Forward 5′-TGACACCCACCTGCAATTTA-3′; Reverse 5′-CAGCACTTCCCACACAAATG-3′; Probe 6-FAM-AAAAAGAAGGATGATTTGCTGC-TAMRA). Relative expression was calculated thanks to the 2^-ΔCt^ method with 18S expression used as the reference gene.

### Public dataset study

The public dataset was downloaded from the Gene Expression Omnibus website. After the selection of genes of interest, the data were log transformed when necessary. Genes and arrays were median centered. Then, non-supervised hierarchical clustering was performed by calculating Euclidian distances.

Statistical analysis was performed with Graph Pad Prism Software or Statview 5.0 software.

## Results

### Expression profile predicting response to docetaxel + transtuzumab-based neoadjuvant chemotherapy

**Figure 1:**
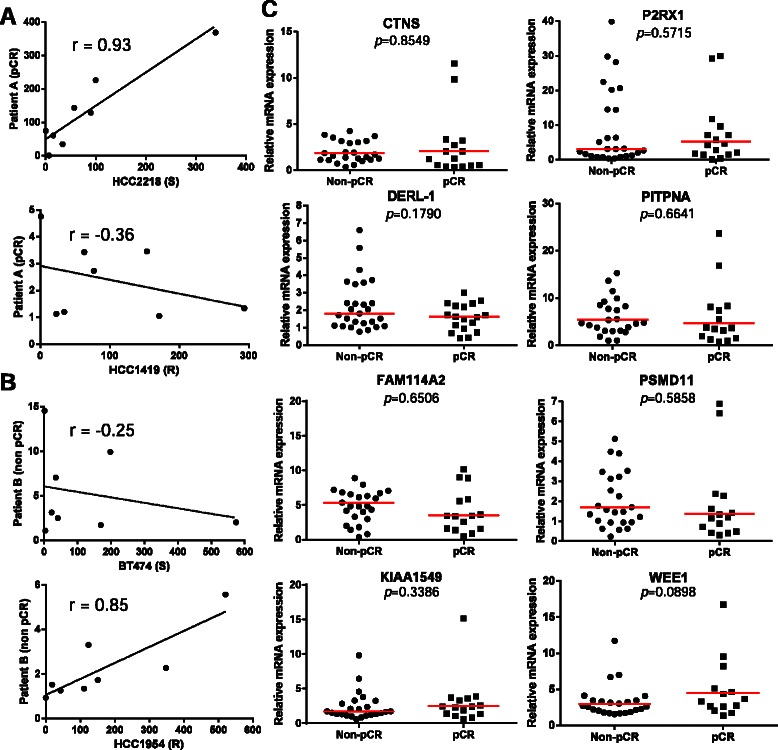
**Data obtained with the analysis of mRNA expression on tumors. A.** Correlation between a pCR tumor with a sensitive (HCC2218) and a resistant (HCC1419) cell line for the expression of the 8 genes. The correlation coefficient for this tumor was 0.93 with sensitive cells and −0.36 with resistant cells, thus classifying it as a sensitive tumor. **B.** Correlation between a non-pCR tumor with a sensitive (BT474) and a resistant (HCC1954) cell line for the expression of the 8 genes. The correlation coefficient for this tumor was −0.25 with sensitive cells and 0.85 with resistant cells, thus classifying it as a resistant tumor. **C.** Expression levels of the 8 genes in pCR and non-pCR tumors. The expression level of each gene was not significantly different between pCR and non-pCR tumors. The *p* value was calculated with the non-parametric Mann and Whitney *U* test. Graphs represent a zoom around the median value, which explains why higher values do not appear on graphs. Median values are indicated by a red solid line.

In order to predict the response to treatment, we calculated the correlation coefficient of each tumor with each cell line. The correlation coefficient nearest to 1 corresponds to the prediction profile. As the tumor response was known, we eliminated genes one by one until we obtained the best prediction performances. This was achieved (Table 2) with the association of the expression of only 8 genes: *CTNS*, *DERL-1*, *FAM114A2*, *KIAA1549*, *P2RX1*, *PITPNA*, *PSMD11*, and *WEE1*. As an example, for patient A with a pCR and patient B with no pCR, the correlation between patient A’s tumor cells and the reference cell lines showed r = 0.93 with the sensitive HCC2218 cells, whereas we obtained r = −0.36 with the resistant HCC1419 cells, classifying it as a sensitive tumor (Figure 1A). In contrast, patient B’s tumor cells correlated positively with the resistant HCC1954 cell line (r = 0.85) and negatively with the sensitive BT474 cells (r = −0.25), classifying it as a resistant tumor (Figure 1B). All correlation coefficients are presented in Additional file 2. Surprisingly, the expression level of these 8 genes individually was not significantly different between pCR patients and non-pCR patients (Figure 1C), suggesting that the combination of the expressions more than the expression of each gene individually was responsible for the prediction capacity. In parallel, we also studied corresponding FFPE samples for 34 patients of our population for expression of the 8 genes. To determine whether our signature could also be assessed on FFPE samples, we calculated correlation coefficients of each gene between frozen and FFPE paired samples. Except for DERL-1 (r = −0.19), the 7 genes CTNS (r = 0.77), FAM114A2 (r = 0.19), KIAA1549 (r = 0.61), P2RX1 (r = 0.44), PITPNA (r = 0.44), PSMD11 (r = 0.39), and WEE1 (r = 0.13) correlated positively suggesting that paraffin treatment of samples did not alter the expression of the genes. The absence of a positive correlation for DERL-1 expression between frozen and FFPE samples could have been due to the smaller number of FFPE samples.

**Table 2:** **Best prediction performances with only 8 genes**

	*Predicted*		
	pCR	Non-pCR	Total
***Observed***			
**pCR**	12	6	18
**Non-pCR**	5	22	27
**Total**	17	28	45
	**Cases**	**Percentage**	
**Sensitivity**	12/18	67	
**Specificity**	22/27	82	
**Positive predictive value**	17/18	94	
**Negative predictive value**	28/27	104	
**Accuracy**	34/45	**76**

### The expression profile is specific to response to first-line neoadjuvant docetaxel + trastuzumab-based chemotherapy

**Figure 2:**
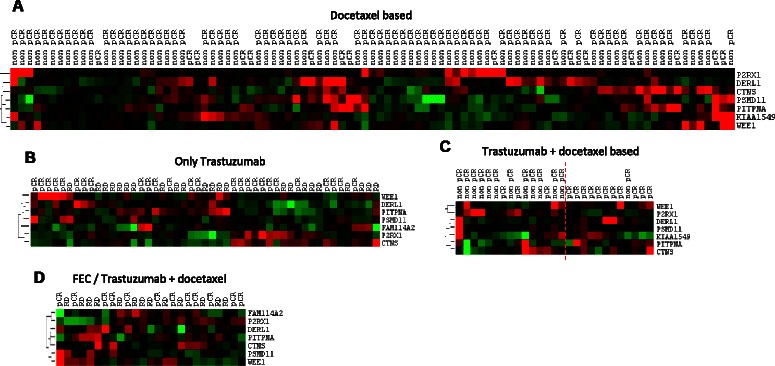
**Non-supervised hierarchical clustering obtained with public datasets. A.** The combined expression of the profile genes did not correctly distinguish between pCR and non-pCR tumors treated with a docetaxel-based chemotherapy. **B.** The same observation was made with a trastuzumab monotherapy regimen. **C.** In contrast, pCR were distinguished from non-pCR tumors (accuracy of 85%) when tumors were treated with a first-line neoadjuvant trastuzumab + docetaxel-based regimen. The vertical red dashed line represents the separation between the 2 response subgroups. **D.** The use of first-line neoadjuvant chemotherapy before treatment with trastuzumab + docetaxel altered the prediction capacity of our profile. Green and red colors represent underexpression or overexpression centered on median array values, respectively.

To test the prediction capacity of the combined expression of the 8 genes in different conditions, we used microarray datasets available on the GEO website. We first correlated the expression of our 8 genes with the expression of *ERBB2*. The 8 genes correlated significantly and positively with ERBB2 expression (Additional file 3). As our first gene expression signature was obtained from tumors treated with trastuzumab + docetaxel-based chemotherapy, we tested the prediction in patients treated with first-line neoadjuvant - docetaxel-based regimen (GSE22358), first-line neoadjuvant - trastuzumab monotherapy (GSE37946), or first-line neoadjuvant - trastuzumab + docetaxel-based chemotherapy (GSE22358). When the regimen contained docetaxel without trastuzumab (Figure 2A) or trastuzumab alone (Figure 2B), our profile was not able to dichotomize tumor response. In contrast, the response of patients treated with first-line trastuzumab + docetaxel-based chemotherapy was well classified by our profile (Figure 2C). Indeed, the accuracy of the classification was 85% (23/27), with a sensitivity of 92% (11/12) and a specificity of 80% (12/15). Finally, it appeared that the profile was not usable for a second-line neoadjuvant trastuzumab + docetaxel-based regimen, at least after first-line neoadjuvant 5-fluorouracile + epirubicin + cyclophosphamide (GSE42822) (Figure 2D). This could be explained by modifications in tumor cell gene expression induced by the first treatment, which may have influenced the subsequent response of tumor cells to docetaxel + trastuzumab.

## Discussion

Based on available public datasets, it appeared that the combination of the expression of only 8 genes could correctly dichotomize the response of HER2-positive advanced breast tumors to first-line trastuzumab + docetaxel-based chemotherapy. The accuracy of prediction was between 76% based on quantitative PCR data (Table 2) and 85% based on the GEO dataset. This is equivalent to the prediction accuracy obtained with the analysis by Positron Emission Tomography of the ^18^FDG uptake of tumors before treatment and after one course of chemotherapy [8,9]. The gene expression method, however, has the advantages of a lower cost of analysis and a prediction available before the therapeutic decision.

The association of trastuzumab and docetaxel is also used as an adjuvant treatment for operable breast cancer [10], and in a small number of non-small-cell lung carcinomas [11]. In these cases, it could be interesting to evaluate the ability of our profile to predict the efficacy of adjuvant chemotherapy in breast cancer and the response of non-operable NSCLC by using activated HER2 lung cancer cell lines as the reference to avoid tissue-origin bias. Recently, HER2 overexpression was detected in 16% of gastric cancers and was associated with a poor prognosis [12]. As this sub-population of gastric cancer patients could benefit from a trastuzumab + docetaxel-based regimen [13], it would be interesting to assess the prediction accuracy of our 8-gene expression profile in this population. Equally, our profile could be tested for the response prediction to a treatment with new HER2 tyrosine kinase inhibitors, which can be associated with docetaxel [14], by using HER2 overexpressing gastric cancer cell lines as the reference.

## Conclusions

**Figure 3:**
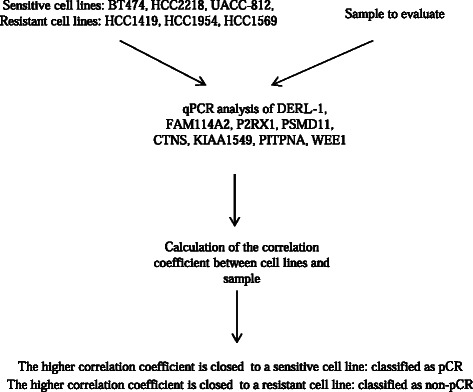
**Methodology for the prediction of pCR or non-pCR.** Six cancer cell lines and tissue samples to analyze were treated in the same way. After real time quantitative PCR analysis, correlation coefficients between samples and cell lines were calculated. The sample of interest was classified as pCR if the higher coefficient was close to a sensitive cell line or as non-pCR if the higher coefficient was close to a resistant cell line.

In conclusion, we showed that analysis of the transcriptional expression of 8 genes present in frozen or FFPE tumors could be used to dichotomize HER2+++ patients as potentially sensitive or resistant to neoadjuvant trastuzumab + docetaxel based chemotherapy (Figure 3). The use of cancer cell lines treated in exactly the same way as tumor cells enables the easy and accurate classification of patients.

## Additional files

Additional file 1:
**Validation of sensitivity and resistance of cancer cell lines used as reference.**

Additional file 2:
**Correlation coefficients obtained for each sample.**

Additional file 3:
**Correlation coefficients between expression of the 8 genes and** ***Erbb2*** **.**

## Abbreviations

HER2: Human epidermal growth factor receptor 2
pCR: Pathological complete response
FFPE: Formalin fixed, paraffin embedded
RD: Residual disease
PCR: Polymerase chain reaction
^18^FDG: 2-desoxy-2-(18 F)fluoro-D-glucose
