# Supplementary material for: Transcriptional expression of 8 genes predicts pathological response to first-line docetaxel + trastuzumab-based neoadjuvant chemotherapy
Source: BMC Cancer. 2015 Mar 24;15:169. doi: 10.1186/s12885-015-1198-9 (PMC4417290; doi:10.1186/s12885-015-1198-9)
Supplement: Additional file 1: — Validation of sensitivity and resistance of cancer cell lines used as reference. [file 12885_2015_1198_MOESM1_ESM.pdf]

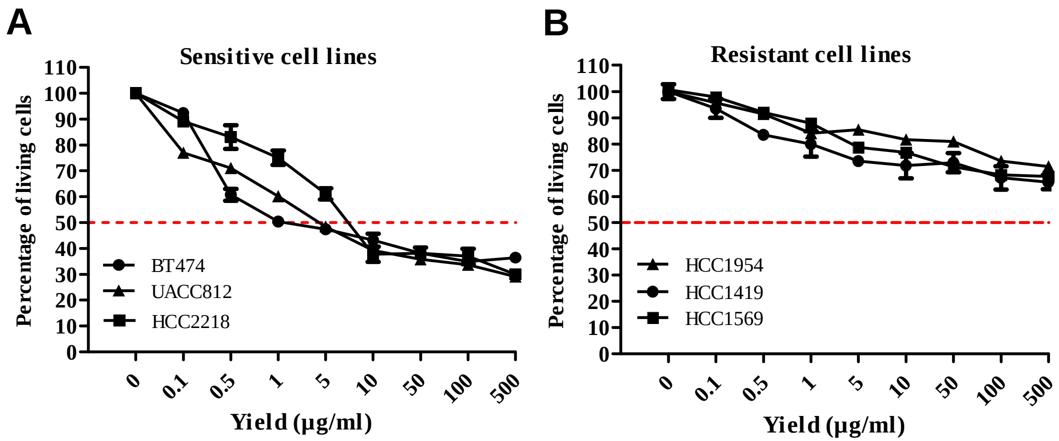

Additional file 2

Additional File 2: Characterization of cell line sensitivity to trastuzumab

The sensitivity of BT474, HCC2218, UACC-812, HCC1419, HCC1954, and HCC1569 cell lines was analyzed with increasing doses of trastuzumab (from 0 to 500 µg/ml). Among the 6 cell lines, -**A-** BT474, UACC-812, and HCC2218 were sensitive to trastuzumab (IC50 = 1µg/ml, 5µg/ml, and 8µg/ml, respectively) and **-B-** the 3 others were resistant (IC50 > 500µg/ml). IC50 is shown by the red dashed line.
