# Supplementary material for: Transcriptional expression of 8 genes predicts pathological response to first-line docetaxel + trastuzumab-based neoadjuvant chemotherapy
Source: BMC Cancer. 2015 Mar 24;15:169. doi: 10.1186/s12885-015-1198-9 (PMC4417290; doi:10.1186/s12885-015-1198-9)
Supplement: Additional file 2: — Correlation coefficients obtained for each sample. [file 12885_2015_1198_MOESM2_ESM.pdf]

Additional File 2: Correlation coefficients obtained for each sample

Patient #	Response	Sensitive BT474	Sensitive HCC2218	Sensitive UACC-812	Resistant HCC1419	Resistant HCC1954	Resistant HCC1569	Prediction	Discordance
1	pCR	0.04004	0.06877	0.40700	0.04674	0.39591	0.38195	Sensitive	No
2	non pCR	-0.04530	-0.06816	-0.23205	-0.21415	-0.05503	-0.01444	Resistant	No
3	non pCR	0.43902	0.38675	0.75903	0.53401	0.85581	0.85938	Resistant	No
4	non pCR	0.29205	0.32921	0.16184	0.25515	0.42464	0.40580	Resistant	No
5	pCR	0.38109	0.37936	0.02377	0.22267	0.29696	0.31572	Sensitive	No
6	pCR	0.08339	0.03969	0.10493	0.01341	0.25179	0.29458	Resistant	Yes
7	pCR	0.30998	0.35352	0.42382	0.32685	0.64033	0.59157	Resistant	Yes
8	non pCR	-0.22866	-0.24229	-0.16604	-0.34744	-0.11324	-0.07798	Resistant	No
9	pCR	0.92538	0.92761	0.44639	0.90216	0.78808	0.76504	Sensitive	No
10	non pCR	0.14806	0.13935	0.48320	0.11080	0.58735	0.60093	Resistant	No
11	pCR	0.18148	0.18658	-0.36346	0.14141	-0.14706	-0.17048	Sensitive	No
12	pCR	0.21873	0.20337	0.85752	0.32159	0.79264	0.78869	Sensitive	No
13	pCR	-0.03046	0.01892	0.45169	0.07231	0.28713	0.23420	Sensitive	No
14	non pCR	0.31264	0.25272	0.64901	0.34127	0.69736	0.74656	Resistant	No
15	non pCR	0.11571	0.08547	-0.54448	-0.10894	-0.19494	-0.13648	Sensitive	Yes
16	non pCR	0.63469	0.62601	-0.03014	0.54418	0.40453	0.41721	Sensitive	Yes
17	pCR	0.60006	0.58750	0.61276	0.55850	0.72292	0.73902	Resistant	Yes
18	non pCR	0.18761	0.18534	0.84955	0.34017	0.75936	0.73061	Sensitive	Yes
19	non pCR	0.26965	0.25727	0.79409	0.35624	0.84622	0.83652	Resistant	No
20	pCR	-0.03281	-0.08267	0.00314	-0.08338	0.06318	0.10519	Resistant	Yes
21	non pCR	0.17691	0.21051	0.54413	0.25190	0.55859	0.50532	Resistant	No
22	non pCR	0.12456	0.19407	0.18599	0.18562	0.30071	0.19613	Resistant	No
23	non pCR	0.16894	0.15401	0.52835	0.28438	0.52731	0.52498	Sensitive	Yes
24	non pCR	0.67540	0.68346	0.72218	0.73048	0.88343	0.85470	Resistant	No
25	non pCR	0.57339	0.59725	0.67220	0.59573	0.85307	0.82302	Resistant	No
26	pCR	-0.32648	-0.32339	0.18971	-0.36140	-0.01247	0.02270	Sensitive	No
27	pCR	0.46019	0.44801	0.05162	0.38183	0.42337	0.43863	Sensitive	No
28	pCR	0.85864	0.84531	0.10792	0.73693	0.52585	0.52820	Sensitive	No
29	pCR	0.73884	0.74019	-0.28279	0.54666	0.23093	0.24299	Sensitive	No
30	pCR	0.91856	0.93028	-0.01449	0.77419	0.46359	0.45198	Sensitive	No
31	non pCR	-0.24730	-0.26466	0.10136	-0.32116	0.08631	0.11908	Resistant	No
32	non pCR	-0.31109	-0.32087	-0.19537	-0.43579	-0.16406	-0.13098	Resistant	No
33	pCR	0.71790	0.72608	0.59395	0.70185	0.87310	0.85790	Resistant	Yes
34	non pCR	-0.19448	-0.19059	-0.20832	-0.33145	-0.16302	-0.13724	Resistant	No
35	non pCR	0.02117	0.05128	0.46055	0.08384	0.44397	0.40652	Sensitive	Yes
36	non pCR	0.28708	0.24290	0.74978	0.42322	0.77357	0.77726	Resistant	No
37	non pCR	-0.22540	-0.25981	-0.03351	-0.32154	0.00137	0.05159	Resistant	No
38	non pCR	0.25099	0.31722	0.29107	0.23424	0.44468	0.40481	Resistant	No
39	non pCR	0.25180	0.22349	0.57761	0.22931	0.64618	0.67387	Resistant	No
40	pCR	0.88449	0.88924	0.33181	0.92555	0.66765	0.62788	Resistant	Yes
41	non pCR	-0.19829	-0.21529	-0.07428	-0.31066	0.01050	0.04862	Resistant	No
42	non pCR	0.10543	0.04178	0.16638	0.26492	0.17246	0.16640	Resistant	No
43	non pCR	0.16120	0.26058	0.24085	0.15093	0.33545	0.25054	Resistant	No
44	pCR	0.68276	0.69102	0.31693	0.64211	0.65749	0.64597	Sensitive	No
45	non pCR	0.40774	0.43977	0.65796	0.46236	0.75265	0.72027	Resistant	No

The underlined coefficient corresponds to the highest, predicting pCR or non-pCR
