# Supplementary material for: Transcriptional expression of 8 genes predicts pathological response to first-line docetaxel + trastuzumab-based neoadjuvant chemotherapy
Source: BMC Cancer. 2015 Mar 24;15:169. doi: 10.1186/s12885-015-1198-9 (PMC4417290; doi:10.1186/s12885-015-1198-9)
Supplement: Additional file 3: — Correlation coefficients between expression of the 8 genes and Erbb2 . [file 12885_2015_1198_MOESM3_ESM.pdf]

Additional file 3: Correlation coefficients between expression of the 8 genes and *Erbb2*

Genes	Correlation coefficient	*p*
DERL1	0.9	< 0.0001
WEE1	0.87	< 0.0001
PITPNA	0.893	< 0.0001
FAM114A2	0.236	0.0048
P2RX1	0.452	< 0.0001
PSMD11	0.898	< 0.0001
CTNS	0.894	< 0.0001
KIAA1549	0.222	0.0137
